# Diazido­bis[(1-methyl-1*H*-benzimidazol-2-yl)methanol-κ^2^ *N* ^3^,*O*]manganese(II)

**DOI:** 10.1107/S1600536810002126

**Published:** 2010-01-23

**Authors:** Yan-Ling Zhou, Hong Liang, Ming-Hua Zeng

**Affiliations:** a School of Chemistry and Chemical Engineering, Central South University, Changsha 410083, People’s Republic of China; b School of Chemistry and Chemical Engineering, Guangxi Normal University, Guilin 541004, People’s Republic of China

## Abstract

The title complex, [Mn(N_3_)_2_(C_9_H_10_N_2_O)_2_], possesses crystallographically imposed twofold symmetry. The Mn^II^ atom is coordinated by four N atoms and two O atoms in a distorted octa­hedral geometry. The crystal packing is stabilized by strong inter­molecular O—H⋯N hydrogen bonds.

## Related literature

For the synthesis of the ligand, see: van Albada *et al.* (1995 ▶) and literature cited therein. For the metal(II) complexes of a similar *N*-heterocycle, see: Zeng *et al.* (2006 ▶); Zhou *et al.* (2007 ▶); Alagna *et al.* (1984 ▶); Hamilton *et al.* (1979 ▶).
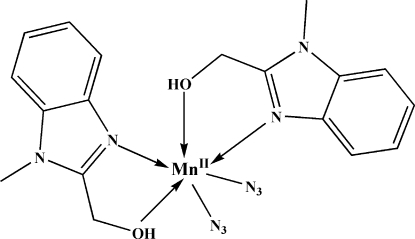


## Experimental

### 

#### Crystal data

[Mn(N_3_)_2_(C_9_H_10_N_2_O)_2_]
*M* *_r_* = 463.38
Monoclinic, 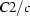

*a* = 15.466 (3) Å
*b* = 7.5438 (16) Å
*c* = 18.095 (4) Å
β = 109.989 (4)°
*V* = 1984.0 (7) Å^3^
*Z* = 4
Mo *K*α radiation
μ = 0.71 mm^−1^
*T* = 173 K
0.33 × 0.22 × 0.10 mm

#### Data collection

Bruker SMART APEX CCD area-detector diffractometer
Absorption correction: multi-scan *SADABS* (Sheldrick, 1996 ▶) *T*_min_ = 0.801, *T*_max_ = 0.933
4125 measured reflections
1741 independent reflections
1345 reflections with *I* > 2σ(*I*)
*R*_int_ = 0.029

#### Refinement

*R*[*F* ^2^ > 2σ(*F* ^2^)] = 0.042
*wR*(*F* ^2^) = 0.116
*S* = 1.02
1741 reflections
142 parameters
H-atom parameters constrained
Δρ_max_ = 0.46 e Å^−3^
Δρ_min_ = −0.29 e Å^−3^

### 

Data collection: *SMART* (Bruker, 2001 ▶); cell refinement: *SAINT* (Bruker, 2001 ▶); data reduction: *SAINT*; program(s) used to solve structure: *SHELXS97* (Sheldrick, 2008 ▶); program(s) used to refine structure: *SHELXL97* (Sheldrick, 2008 ▶); molecular graphics: *X-SEED* (Barbour, 2001 ▶); software used to prepare material for publication: *publCIF* (Westrip, 2010 ▶) and *PLATON* (Spek, 2009 ▶).

## Supplementary Material

Crystal structure: contains datablocks global, I. DOI: 10.1107/S1600536810002126/si2238sup1.cif

Structure factors: contains datablocks I. DOI: 10.1107/S1600536810002126/si2238Isup2.hkl

Additional supplementary materials:  crystallographic information; 3D view; checkCIF report

## Figures and Tables

**Table 1:** Selected bond lengths (Å)

O1—Mn1	2.302 (2)
Mn1—N3	2.172 (3)
Mn1—N1	2.176 (2)

Symmetry code: (i) 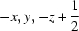.

**Table 2:** Hydrogen-bond geometry (Å, °)

*D*—H⋯*A*	*D*—H	H⋯*A*	*D*⋯*A*	*D*—H⋯*A*
O1—H1⋯N5^ii^	0.85	1.85	2.701 (4)	178

Symmetry code: (ii) 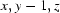.

**Fig. 1.**
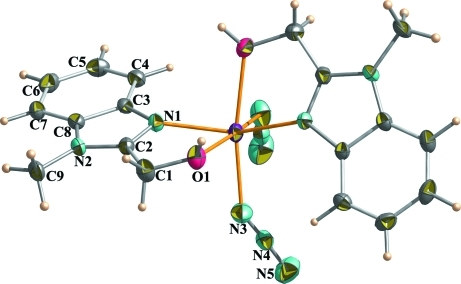
Anisotropic displacement ellipsoid plot of the [Mn(II)(N3)2(C9H10N2O)2] molecule at the 50% probability level; hydrogen atoms are drawn as sphere of arbitrary radius. Symmetry codes: (i) -x, y, -z + 1/2, for the unlabelled atoms.

**Fig. 2.**
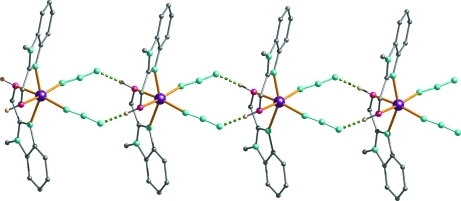
Part of the hydrogen bonded chain along [010] direction. Hydrogen bonds are shown as dashed lines. Symmetry codes: (i) x, y - 1, z.

## supplementary crystallographic information

### Comment

The coordinated modes of (1-methyl-1*H*-benzimidazol-2-yl)methanol ligand are similar to our previously repored benzimidazol-2-yl methanol from the structural point, the latter has been shown to bind to cobalt(II) as a neutral chelate (Zeng *et al.*, 2006, Zhou *et al.*, 2007). This feature is also preserved in the present manganese(II) complex.

In the title compound, the ligand chelates through the hydroxyl O and imino N atoms, resulting in a N_4_O_2_Mn octahedral geometry at the metal center (Fig. 1, Table 1), like that observed in copper (Hamilton *et al.*, 1979) and nickel (Alagna *et al.*, 1984) adducts. In this structure, the azide anion as a terminal ligand coordinated to Mn^II^ atom, and N–N–N bond lengths and bond angle are close to compound [Cu(tbz)(N_3_)_2_]_2_(CH_3_OH)_2_ (tbz = bis(2-benzimidazolyl)propane) (Albada *et al.*, 1995).The complex possesses crystallographically imposed twofold symmetry. The crystal packing is stabilized by strong intermolecular O—H···N hydrogen bonds which extend along the crystallographic twofold rotation axis (Fig. 2, Table 2).

### Experimental

(1-methyl-1*H*-benzimidazol-2-yl)methanol was purchased from a chemical supplier. This reagent (0.16 g, 1 mmol), manganese(II) nitrate hexahydrate (0.14 g, 0.5 mmol) and sodium azide (0.07 g, 1 mmol) were dissolved in water (10 ml) that was kept at about 333 K. Colorless blocks separated from the solution after one week.

### Refinement

The C-bound H atoms were placed in calculated positions (C—H = 0.93–0.98 Å) and included in the refinement in the riding-model approximation, with *U*_iso_(H) = 1.2(1.5)*U*_eq_(C, C_methyl_). The hydroxy H atom has been located in a difference Fourier map and refined isotropically with a distance restraint of O—H = 0.85 (1) Å, and *U*_iso_(H) = 1.2*U*_eq_(O).

### Crystal data

**Table:**

[Mn(N_3_)_2_(C_9_H_10_N_2_O)_2_]	*F*(000) = 956
*M**_r_* = 463.38	*D*_x_ = 1.551 Mg m^−^^3^
Monoclinic, *C*2/*c*	Mo *K*α radiation, λ = 0.71073 Å
Hall symbol: -C 2yc	Cell parameters from 1818 reflections
*a* = 15.466 (3) Å	θ = 2.4–26.7°
*b* = 7.5438 (16) Å	µ = 0.71 mm^−^^1^
*c* = 18.095 (4) Å	*T* = 173 K
β = 109.989 (4)°	Block, colorless
*V* = 1984.0 (7) Å^3^	0.33 × 0.22 × 0.10 mm
*Z* = 4	

### Data collection

**Table:**

Bruker SMART APEX CCD area-detector diffractometer	1741 independent reflections
Radiation source: fine-focus sealed tube	1345 reflections with *I* > 2σ(*I*)
graphite	*R*_int_ = 0.029
phi and ω scans	θ_max_ = 25.0°, θ_min_ = 2.8°
Absorption correction: multi-scan *SADABS* (Sheldrick, 1996)	*h* = −16→18
*T*_min_ = 0.801, *T*_max_ = 0.933	*k* = −8→8
4125 measured reflections	*l* = −15→21

### Refinement

**Table:**

Refinement on *F*^2^	Primary atom site location: structure-invariant direct methods
Least-squares matrix: full	Secondary atom site location: difference Fourier map
*R*[*F*^2^ > 2σ(*F*^2^)] = 0.042	Hydrogen site location: inferred from neighbouring sites
*wR*(*F*^2^) = 0.116	H-atom parameters constrained
*S* = 1.02	*w* = 1/[σ^2^(*F*_o_^2^) + (0.0608*P*)^2^ + 4.1072*P*] where *P* = (*F*_o_^2^ + 2*F*_c_^2^)/3
1741 reflections	(Δ/σ)_max_ < 0.001
142 parameters	Δρ_max_ = 0.46 e Å^−^^3^
0 restraints	Δρ_min_ = −0.29 e Å^−^^3^

### Special details

**Table:**

Refinement. Refinement of *F*^2^ against ALL reflections. The weighted *R*-factor *wR* and goodness of fit *S* are based on *F*^2^, conventional *R*-factors *R* are based on *F*, with *F* set to zero for negative *F*^2^. The threshold expression of *F*^2^ > σ(*F*^2^) is used only for calculating *R*-factors(gt) *etc*. and is not relevant to the choice of reflections for refinement. *R*-factors based on *F*^2^ are statistically about twice as large as those based on *F*, and *R*- factors based on ALL data will be even larger.In Checkcif report, the following ALERTS were generatedPLAT230_ALERT_2_C Hirshfeld Test Diff for N3 – N4.. 5.98 su Author response: It is due to electron shift or resonance (N=N–N or N–N=N) bond lengths appear shorter than expected, see: Albada *et al.* (1995).

### Fractional atomic coordinates and isotropic or equivalent isotropic displacement parameters (Å^2^)

**Table:**

	*x*	*y*	*z*	*U*_iso_*/*U*_eq_	
O1	0.12170 (15)	0.1356 (3)	0.26588 (12)	0.0324 (5)	
H1	0.1264	0.0343	0.2875	0.049*	
Mn1	0.0000	0.32504 (9)	0.2500	0.0247 (2)	
N1	−0.00030 (17)	0.2562 (3)	0.13315 (13)	0.0228 (6)	
N2	0.07803 (16)	0.1833 (3)	0.05480 (14)	0.0226 (5)	
N3	0.1101 (2)	0.5198 (4)	0.28397 (17)	0.0405 (7)	
N4	0.11891 (18)	0.6678 (4)	0.30523 (15)	0.0324 (7)	
N5	0.1360 (3)	0.8099 (4)	0.3311 (2)	0.0545 (9)	
C1	0.1524 (2)	0.1270 (5)	0.20037 (18)	0.0316 (8)	
H1A	0.1698	0.0038	0.1928	0.038*	
H1B	0.2070	0.2037	0.2096	0.038*	
C2	0.0761 (2)	0.1879 (4)	0.12911 (17)	0.0226 (6)	
C3	−0.05362 (19)	0.2995 (4)	0.05571 (17)	0.0208 (6)	
C4	−0.1407 (2)	0.3733 (4)	0.02570 (18)	0.0257 (7)	
H4A	−0.1745	0.4034	0.0590	0.031*	
C5	−0.1764 (2)	0.4013 (4)	−0.05442 (18)	0.0307 (7)	
H5A	−0.2359	0.4520	−0.0767	0.037*	
C6	−0.1269 (2)	0.3569 (4)	−0.10337 (18)	0.0331 (8)	
H6A	−0.1538	0.3782	−0.1583	0.040*	
C7	−0.0403 (2)	0.2830 (4)	−0.07448 (18)	0.0289 (7)	
H7B	−0.0068	0.2526	−0.1080	0.035*	
C8	−0.0045 (2)	0.2554 (4)	0.00618 (17)	0.0230 (6)	
C9	0.1533 (2)	0.1183 (5)	0.03086 (19)	0.0309 (7)	
H9A	0.1617	−0.0089	0.0419	0.046*	
H9B	0.2100	0.1814	0.0602	0.046*	
H9C	0.1387	0.1388	−0.0256	0.046*	

### Atomic displacement parameters (Å^2^)

**Table:**

	*U*^11^	*U*^22^	*U*^33^	*U*^12^	*U*^13^	*U*^23^
O1	0.0388 (13)	0.0343 (13)	0.0242 (11)	0.0082 (10)	0.0111 (10)	0.0071 (9)
Mn1	0.0313 (4)	0.0266 (4)	0.0185 (3)	0.000	0.0116 (3)	0.000
N1	0.0244 (13)	0.0266 (14)	0.0183 (12)	0.0014 (11)	0.0087 (10)	−0.0004 (10)
N2	0.0221 (13)	0.0260 (13)	0.0238 (12)	0.0008 (10)	0.0129 (10)	−0.0026 (10)
N3	0.0522 (19)	0.0308 (18)	0.0451 (18)	−0.0144 (14)	0.0254 (15)	−0.0073 (14)
N4	0.0325 (15)	0.043 (2)	0.0243 (14)	−0.0057 (14)	0.0135 (12)	0.0027 (13)
N5	0.081 (3)	0.0340 (19)	0.048 (2)	−0.0135 (18)	0.0225 (19)	−0.0063 (16)
C1	0.0296 (17)	0.0375 (19)	0.0296 (17)	0.0092 (14)	0.0125 (14)	0.0043 (14)
C2	0.0248 (16)	0.0230 (16)	0.0214 (14)	−0.0001 (13)	0.0096 (12)	−0.0014 (12)
C3	0.0216 (15)	0.0198 (15)	0.0221 (14)	−0.0029 (12)	0.0088 (12)	0.0016 (11)
C4	0.0231 (16)	0.0266 (17)	0.0295 (16)	−0.0012 (13)	0.0117 (13)	−0.0016 (13)
C5	0.0238 (16)	0.0314 (18)	0.0314 (17)	0.0006 (14)	0.0023 (14)	0.0036 (14)
C6	0.0376 (19)	0.036 (2)	0.0205 (15)	−0.0087 (15)	0.0031 (14)	0.0024 (13)
C7	0.0333 (18)	0.0334 (19)	0.0235 (15)	−0.0070 (14)	0.0140 (14)	−0.0023 (13)
C8	0.0233 (15)	0.0240 (15)	0.0237 (15)	−0.0033 (12)	0.0106 (12)	−0.0022 (12)
C9	0.0273 (17)	0.0376 (19)	0.0330 (17)	0.0039 (14)	0.0170 (14)	−0.0043 (14)

### Geometric parameters (Å, °)

**Table:**

O1—C1	1.421 (4)	C1—H1A	0.9900
O1—Mn1	2.302 (2)	C1—H1B	0.9900
O1—H1	0.8500	C3—C4	1.385 (4)
Mn1—N3	2.172 (3)	C3—C8	1.399 (4)
Mn1—N3^i^	2.172 (3)	C4—C5	1.380 (4)
Mn1—N1	2.176 (2)	C4—H4A	0.9500
Mn1—N1^i^	2.176 (2)	C5—C6	1.395 (5)
Mn1—O1^i^	2.302 (2)	C5—H5A	0.9500
N1—C2	1.314 (4)	C6—C7	1.379 (5)
N1—C3	1.400 (4)	C6—H6A	0.9500
N2—C2	1.355 (4)	C7—C8	1.388 (4)
N2—C8	1.389 (4)	C7—H7B	0.9500
N2—C9	1.459 (4)	C9—H9A	0.9800
N3—N4	1.174 (4)	C9—H9B	0.9800
N4—N5	1.163 (4)	C9—H9C	0.9800
C1—C2	1.492 (4)		
			
C1—O1—Mn1	114.60 (17)	C2—C1—H1B	110.0
C1—O1—H1	110.0	H1A—C1—H1B	108.4
Mn1—O1—H1	123.6	N1—C2—N2	113.0 (3)
N3—Mn1—N3^i^	94.89 (17)	N1—C2—C1	122.2 (3)
N3—Mn1—N1	100.23 (10)	N2—C2—C1	124.8 (3)
N3^i^—Mn1—N1	98.36 (10)	C4—C3—C8	120.8 (3)
N3—Mn1—N1^i^	98.36 (10)	C4—C3—N1	130.3 (3)
N3^i^—Mn1—N1^i^	100.23 (10)	C8—C3—N1	108.8 (3)
N1—Mn1—N1^i^	152.37 (14)	C5—C4—C3	117.4 (3)
N3—Mn1—O1^i^	169.59 (9)	C5—C4—H4A	121.3
N3^i^—Mn1—O1^i^	81.74 (10)	C3—C4—H4A	121.3
N1—Mn1—O1^i^	90.02 (9)	C4—C5—C6	121.4 (3)
N1^i^—Mn1—O1^i^	72.72 (8)	C4—C5—H5A	119.3
N3—Mn1—O1	81.74 (10)	C6—C5—H5A	119.3
N3^i^—Mn1—O1	169.59 (9)	C7—C6—C5	122.0 (3)
N1—Mn1—O1	72.72 (8)	C7—C6—H6A	119.0
N1^i^—Mn1—O1	90.02 (9)	C5—C6—H6A	119.0
O1^i^—Mn1—O1	103.23 (12)	C6—C7—C8	116.5 (3)
C2—N1—C3	105.6 (2)	C6—C7—H7B	121.8
C2—N1—Mn1	116.39 (19)	C8—C7—H7B	121.8
C3—N1—Mn1	136.2 (2)	C7—C8—N2	132.4 (3)
C2—N2—C8	106.9 (2)	C7—C8—C3	121.9 (3)
C2—N2—C9	126.4 (3)	N2—C8—C3	105.7 (2)
C8—N2—C9	126.6 (2)	N2—C9—H9A	109.5
N4—N3—Mn1	136.7 (3)	N2—C9—H9B	109.5
N5—N4—N3	173.4 (4)	H9A—C9—H9B	109.5
O1—C1—C2	108.4 (2)	N2—C9—H9C	109.5
O1—C1—H1A	110.0	H9A—C9—H9C	109.5
C2—C1—H1A	110.0	H9B—C9—H9C	109.5
O1—C1—H1B	110.0		

Symmetry codes: (i) −*x*, *y*, −*z*+1/2.

### Hydrogen-bond geometry (Å, °)

**Table:**

*D*—H···*A*	*D*—H	H···*A*	*D*···*A*	*D*—H···*A*
O1—H1···N5^ii^	0.85	1.85	2.701 (4)	178

Symmetry codes: (ii) *x*, *y*−1, *z*.
